# Photobiomodulation and Miescher’s cheilitis granulomatosa: case report

**DOI:** 10.1186/s40902-020-00279-y

**Published:** 2020-10-20

**Authors:** Massimo Porrini, Umberto Garagiola, Margherita Rossi, Moreno Bosotti, Sonia Marino, Aldo Bruno Giannì, Letterio Runza, Francesco Spadari

**Affiliations:** 1 Department of Biomedical, Surgical and Dental Sciences-Maxillo-Facial and Odontostomatology Unit-Ospedale Maggiore Policlinico, Fondazione IRCCS Ca’ Granda, University of Milan, via della Commenda 10, Milan, Italy; 2 Division of Pathology, Ospedale Maggiore Policlinico, Fondazione IRCCS Ca’ Granda, University of Milan, Milan, Italy

**Keywords:** Diode laser, Photobiomodulation, Miescher granulomatous cheilitis, Orofacial granulomatosis

## Abstract

**Background:**

Miescher’s cheilitis granulomatosa (MCG) is a rare chronic inflammatory disease and is known as the monosymptomatic clinical form of Melkersson-Rosenthal syndrome (MRS). It is characterised by swelling of one or both lips and more frequently affects the upper lip. Histopathological findings show the presence of numerous inflammatory infiltrates and granuloma formations. Pharmacological treatments and surgery have provided results that are positive yet insufficiently stable in the long term. The clinical case described is of a 68-year-old female patient with a diagnosis of MCG of the upper lip.

**Case presentation:**

The patient was diagnosed and treated at the Oral Medicine and Oral Pathology outpatient clinic of Maxillofacial and Odontostomatology Unit, Fondazione Cà Granda IRCCS Ospedale Maggiore Policlinico. The patient was recommended localised treatments of photobiomodulation (PBM) using a diode laser with a 635 nm and 980 nm dual-wavelength (*λ*) approach, a 600-micron fibre, and a handpiece with a 1-cm-diameter lens at 300 mW. Three treatments a week were administered for four weeks for a total of 12 treatment sessions (*T*_1_–*T*_12_). After that, the patient had a long follow-up period of about 2 years. The therapeutic results were clear from the initial stages of treatment. There was an immediate, gradual, and consistent reduction in labial swelling. A reduction in the size of the lip by about 35% at *T*_10_–*T*_12_ was observed, returning the size and volume of the upper lip within the normal clinical range. The painful symptoms subsided after the seventh treatment (*T*_7_). The histopathological check at 3 months and the follow-up in particular confirmed the disease was in remission with satisfactorily stable treatment results. Moreover, the patient did not use any other treatments on the area from the early laser treatments through to the end of the follow-up period.

**Conclusions:**

Our experience describes a clinical case of MCG treated with PBM and effectively resolved with a reduction of the lip swelling. The real success of the treatment emerged over time, showing that the tissue healing was stable. In absence of any collateral phenomena, this confirms the effective and documented therapeutic potential of PBM for chronic inflammatory infiltrates.

## Background

The term granulomatosis indicates a group of diseases which is basically characterised by a pathogenesis of inflammation, a slow, progressive evolution, and a consistent tendency to become chronic. These can be observed as individual lesions, multiple lesions, lesions spread throughout an organ or system or systemic multifocal spread. It is difficult to reach an aetiological, pathogenic, and clinical finding for this group of diseases [1].

Clinical forms are characterised by a systemic multiregional onset and subsequent slow evolution with preferential localisation in a certain region or organ. For example, sarcoidosis and Crohn’s disease [2]. Other clinical forms are characterised from the early stages by localisation and stable evolution in an individual organ, such as foreign body granulomatosis and some forms of chronic dermatosis [3]. A further aetiological classification identifies infectious and non-infectious forms of granulomatosis. The infectious forms of granulomatosis include systemic tuberculosis, organ tuberculosis, and tertiary syphilis. The non-infectious granulomatoses fall within a group of diseases that are rather diverse and difficult to diagnose; they are basically characterised by a slowly progressing immunological and inflammatory pathogenetic matrix against as yet unknown exogenous factors [4, 5]. Finally, granulomatosis can be divided topographically based on the position or region of the body that is affected. Specifically, Authors consider orofacial granulomatosis (OFG) diseases, characterised by individual localised granulomas or the formation of multiple granulomas [6].

The clinical progression of all forms of OFG is typically chronic with possible remissions followed by subsequent flare-ups. In many cases they present with localised lesions and completely asymptomatic. Forms of OFG present clinical, evolutionary and, more importantly, histopathologic features that are remarkably similar to systemic granulomatous diseases, such as Crohn’s disease. However, none of these clinical forms are accompanied by the intestinal symptoms and signs typical of Crohn’s disease. All oral and maxillomandibular regions may potentially be affected. Lesions on the lips, the body of the tongue, oropharynx, palate, and gingival mucosa occur with varying frequency [7]. Although lacking in specific characteristics, these lesions are characterised by the constant swelling of the affected tissues. Under clinical observation oedema, inflammatory infiltrate lesions, granulomas, and erythematous and ulcerated areas may by present. Diagnosis is often difficult and a combination of systemic clinical data, localised signs and symptoms, and microscopic examinations need to carefully correlated [8].

Amongst the non-infectious OFG, we find MRS and its monosymptomatic, clinical variant represented by MCG, which was described for the first time by Miescher in 1945 [9]. MRS is characterised by a clinical triad of oedema on the lips, which rarely even affects parts of the face, a plicated or fissured swollen tongue, and relapsing facial nerve paralysis. At least two of these clinical presentations are required for a diagnosis of MRS [9]. It is considered to be a rare disease and generally shows no particular preference for ethnic background, age, or sex. However, there are some epidemiological data which state that is a slightly higher prevalence in females [10].

MCG typically affects only the lip region and is characterised by chronic, sometimes recurrent, swelling, often in the absence of any other symptoms and affects the lips thickness to varying degrees. The upper lip is more frequently affected, although both lips can be afflicted. Swelling and oedematous thickening often make it difficult to move the lips, consume solid or liquid food, and produce or utter speech sounds. The aesthetic aspects in more severe cases should be considered [11, 12].

In the early stages of MCG, histological characteristics may be non-specific with the presence of oedema, ectasia of arteries, veins and lymphatic vessels, inflammatory angiogenesis, and perivascular infiltrates of lymphocytes, plasma cells and histiocytes. In the later stages of the disease, granuloma formations are present with multinucleate giant cells of the langhans type, without signs of vascular necrosis or caseous necrosis [8]. The presence of granulomas is characteristic, although their absence does not preclude a diagnosis of MCG. Considering that a differential diagnosis applies to some lesions on the lips, a diagnosis of MCG can be correctly formulated by carefully assessing the patient history, the duration of the lesions on the lip, and the correlations of clinical and histopathologic features [11, 12].

Numerous treatment protocols and experiences have been described and documented in the literature. The most commonly described treatments are pharmacological treatments with local, systemic and intralesional steroid therapies. The most commonly used pharmaceuticals are systemic prednisone, intralesional triamcinolone, and combinations of antibiotic and steroid treatment. Immunosuppressive drugs have also been used, including methotrexate, monoclonal antibodies such as infliximab and adalimumab, and systemic antibiotics such as tetracycline and metronidazole. However, all of the treatments that have been documented in scientific literature have had variable immediate results and, above all, are short lasting in the long term. Reductive surgical treatments have shown to have immediate therapeutic success, but even these treatments have shown early and late tendencies towards relapse [13, 14].

This clinical case of MCG was treated with low-level laser therapies (LLLT), which are now more correctly known by the name photobiomodulation (PBM). It has been widely documented in the literature that localised PBM treatments have proven to have significant anti-inflammatory properties, facilitate tissue repair, and control painful symptoms [15, 16].

## Clinical presentation

**Fig. 1:**
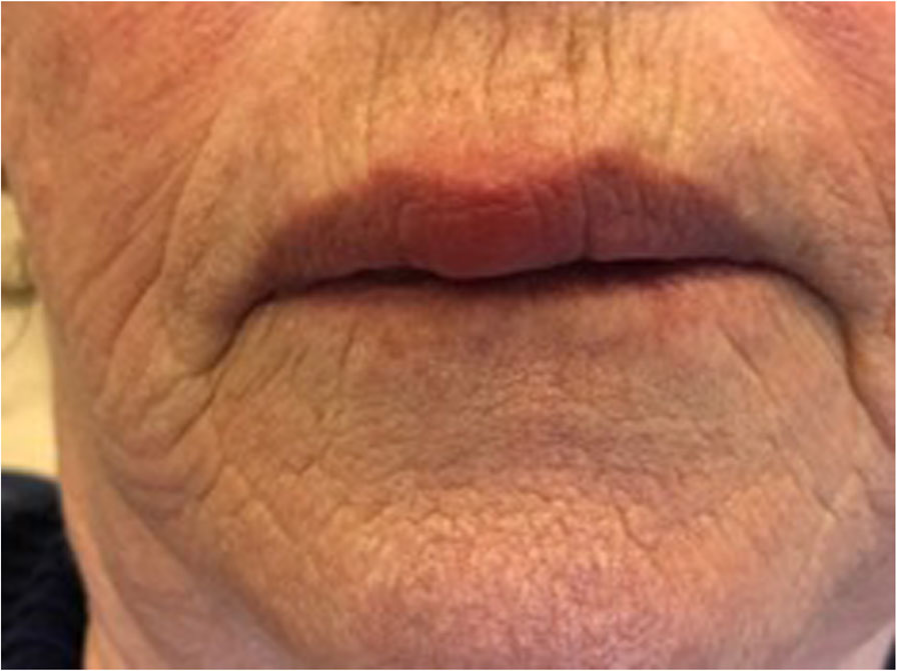
Swelling of the medial portion of the upper lip

A 68-year-old Caucasian woman was referred for observation at the Oral Medicine and Oral Pathology outpatient Clinic of the Maxillofacial and Odontostomatology Unit, Fondazione Cà Granda IRCCS Ospedale Maggiore Policlinico of Milan, in January 2018. The patient presented with swelling of the medial part of the upper lip (Fig. 1), which had been growing slowly and gradually for at least 12 months. However, the patient reported that the swelling of the lip had become more prominent over the past 2 months. By now, the tumefaction affected the breadth and depth of the entire upper lip and vermilion border; it was clearly noticeable at rest and when the lips were moving, and this had just recently caused painful symptoms and some functional limitations.

Systemic diseases were excluded after carefully analysing the medical history. The patient also presented in optimal organic-physical condition in keeping with her age. The patient also reported that her habits and everyday lifestyle were normal, and that she neither drank alcohol nor was a smoker.

During the clinical observation, the swelling of the lip was noticeable with the edges of the oedematous lesion being well defined with regard to the apparently unaffected surrounding tissues. Upon manual palpation, the tissues seemed to have a uniform rubbery consistency without any nodular formations. For the purposes of evaluating the size of the oedema, transverse measurements were taken of the lips with a tape measure and plastic medical callipers to calculate the thickness. At *T*_0,_ the measurement between the labial commissures of the mouth was 9 cm, while the thickness of the lip in the medial zone was 2.7 cm.

According to the medical history, the patient was not taking any pharmaceuticals, had not been in contact with any potentially harmful substances, and was not allergic to any type of substance. Clinical data ruled out an initial diagnostic hypothesis of angioedema. The remaining oral and perioral tissues and the tissues in the neck seemed to be within the normal morphological and functional range without any apparent current pathological conditions.

The patient was discharged with a prescription for further diagnostic procedures. Blood chemistry analysis confirmed organic conditions within the norm without any sign or presence of systemic or local inflammatory and immunological diseases. Ultrasound scans of the perioral area and neck confirmed the presence of localised inflammation without any lymphadenopathy, diseases of the vascular regions, or neoplastic tissue growths. Finally, X-ray examinations revealed that the dental and periodontal health is not particularly impaired and as such compatible with the age of the patient.

Based on all the clinical and instrumental data, the diagnosis was directed towards a form of chronic cheilitis of non-infectious origin but probable responsive and immunological in origin and without a specific, well-defined aetiology. Within the range of possible hypotheses of idiopathic cheilitis, the diagnostic hypothesis of MCG was noteworthy. The lack of other clinical characteristics, such as facial nerve paralysis and macroglossia with plicated tongue, suggested a monosymptomatic form of cheilitis, without the distinctive MRS clinical triad [11, 12].

**Fig. 2:**
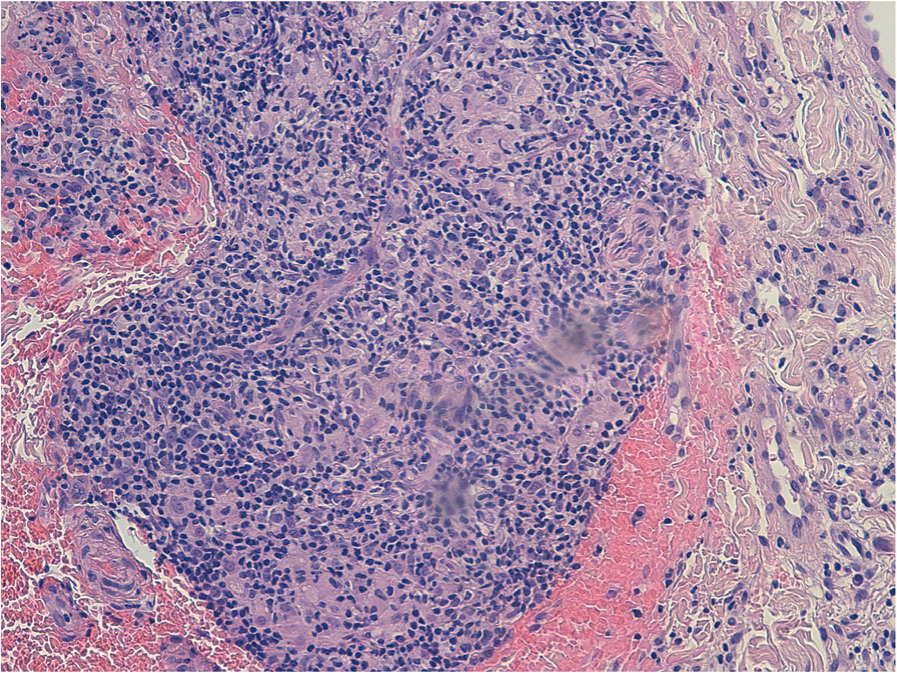
20× (E&E): Chronic epithelioid granulomatous inflammation without necrosis or confluence areas

**Fig. 3:**
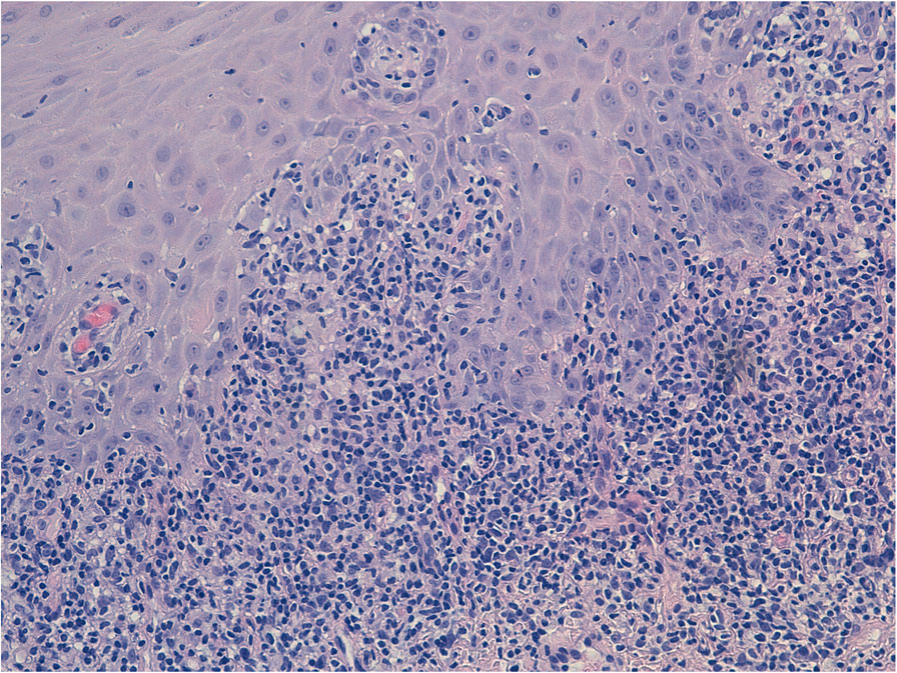
20× (E&E): Polymorphous lymphomonocytic inflammatory infiltrate with affected basal lamina of the oral mucosa

Multiple incisional biopsies were taken from the upper lip to complete diagnosis. Microscopic analysis provided a histopathological report with marked chronic inflammation, inflammatory cell infiltrates, and the formation of granulomas containing epithelioid cells. No residues of necrotic tissue were observed centred in or peripheral to the granulomatous tissues (Figs. 2 and 3). Microscopic analysis confirmed the clinical diagnosis of MCG [4, 8].

Once formulated the diagnosis by properly correlating the clinical and histopathologic data, analysing the treatment options, and sharing our decisions with the patient, we decided to implement a treatment protocol with PBM.

For the purposes of evaluating the size of the oedema, transverse measurements were taken of the lips with a tape measure and plastic medical callipers to calculate the thickness.

Furthermore, a unidimensional scale, the Numeric Pain Rating Scale (NPRS), we used to assess the painful symptoms reported by the patient. A pain score of 3 on the NPRS was assessed at *T*_0_.

**Fig. 4:**
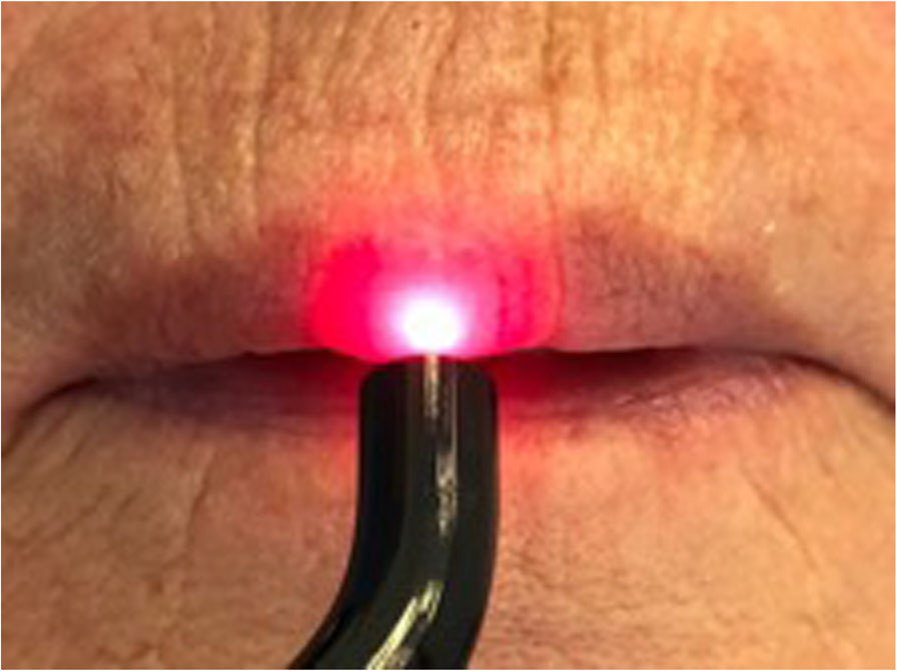
Photobiomodulation therapy using a dual-wavelength diode laser

Treatments began at the end of January 2018 using a diode laser with a 635 nm and 980 nm dual-wavelength (*λ*) approach, a 600 micron fibre, and a handpiece with a 1-cm-diameter lens. The setting was continuous wave (CW) mode at 300 mW with treatment times of 1 min per cm^2^, fluence of 22 J/cm^2^, and defocused mode, with slow, scanning movements in a grid-like pattern (Fig. 4). Treatment times were 10 min a session, 5 min with 635 nm, a 1-min pause and 5 min with 980 nm. Both the mucosal and transdermal laser applications were set to the dual-wavelength in order to achieve PBM with two depth levels. Radiation with *λ* = 635 nm acts on the dense network of type C unmyelinated fibres with intradermal free nerve endings. Radiation with *λ* = 980 nm penetrates to layers which are about 5–6 mm deeper and can also act on type Aδ nerve fibres.

Overall, about 5 cm^2^ of the mucosal and cutaneous areas of the lip were treated. Three PBM treatments a week were administered for 4 weeks. We performed a total of 12 treatments (*T*_1_–*T*_12_). The PBM treatments were concluded at the end of February 2018.

The therapeutic results were clear from the initial stages of treatment, particularly with regard to the painful symptoms which began to improve with the earlier applications (*T*_3_–*T*_4_). Pain in the lip subsided conclusively after the seventh treatment (*T*_7_).

There was also a marked reduction in labial swelling which was gradual yet consistent throughout all stages of the treatment. a reduction in the size of the lip by about 35% Was observed at *T*_10_–*T*_12_ compared with the initial measurements recorded T_0._

## Conclusions

PBM is currently intended as a form of non-thermal local light therapy that can trigger photophysical and photochemical effects at different levels in living matter. Thus, these effects have been shown in different types of body tissues and in cellular structures, particularly at the cytoplasmic level, and in the energy-producing mitochondrial corpuscles. PBM has proven to be effective in reducing inflammatory cytokines, such as prostaglandin E_2_ (PGE_2_) and cyclooxygenase-2 (COX-2), and suppressing interleukin-1β (IL1β) and tumour necrosis factor α (TNFα) [17, 18]. Furthermore, PBM can trigger a significant increase in tissue of endothelial growth factors. These properties should be viewed as therapeutic as they promote increased immune responses with significant effects in terms of decreasing inflammation, repairing damaged tissue, and producing analgesia [19–21].

**Fig. 5:**
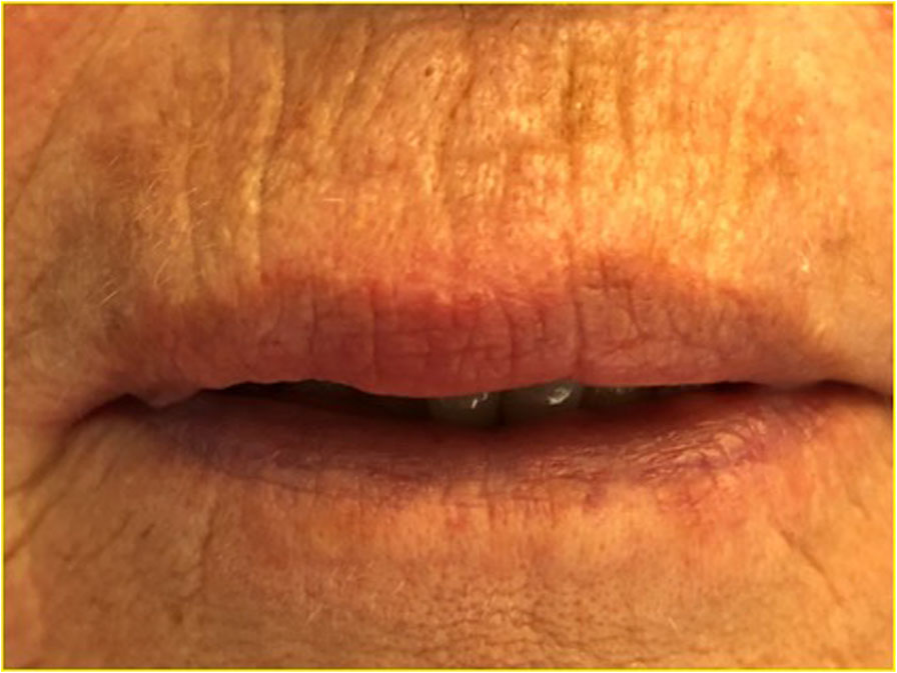
Clinical result achieved after photobiomodulation with LLLT which remained stable over the next 2 years

PBM treatments have produced significant reduction of the lip oedema, with reduced transverse measurements and reduced thickness with values close to 35%, returning the size and volume of the upper lip within the normal clinical range. The subsequent clinical checks scheduled every 15 days for 4 months and monthly follow-ups for about 2 years after enabled to monitor and check the results of the treatment. A second biopsy performed 3 months after the end of the PBM applications also enabled us to check and confirm that the treated tissues were healing, without any inflammation, inflammatory angiogenesis was reduced and the granuloma formations were smaller in size. Over the course of the 2-year follow-up period, there was no sign whatsoever that the disease was returning and no localised inflammation (Fig. 5). Furthermore, during the entire period of treatment and subsequent follow-ups, the patient never took any local or systemic anti-inflammatory steroid medication that could have interfered with the healing process.

The experiences of the authors with laser treatments and the presented clinical case confirm that a careful, and informed PBM treatment programme can ensure results and levels of healing that are optimal and, more importantly, stable in the long term. The present case report is an interesting starting point for further in-depth analyses and researches on the efficacy of the proposed treatment; indeed, although promising, it cannot be judged based on a single case.

Thus, these results could direct future treatment choices towards also using localised PBM treatments for other types of chronic inflammatory diseases that affect the mucosae and oral and maxillomandibular tissues and which are often resistant to pharmacological treatments.

Furthermore, it is important to add that PBM treatments have proven to be free from adverse and collateral effects which means that they would more recommendable and suitable for the elderly and patients undergoing long-term multidrug therapy.

## Data Availability

Non applicable

## Abbreviations

MCG: Miescher’s cheilitis granulomatosa
MRS: Melkersson-Rosenthal syndrome
PBM: Photobiomodulation
OFG: Orofacial granulomatosis
LLLT: Low-level laser therapies
NPRS: Numeric Pain Rating Scale
CW: Continuous wave
PGE2: Prostaglandin E2
COX-2: Cyclooxygenase-2
IL1β: Interleukin-1β
TNFα: Tumour necrosis factor α
